# The Effect of Absorbed Hydrogen on the Rotors of Steel Machining Products During Powerful Turbo Aggregate Repairs

**DOI:** 10.3390/ma17246257

**Published:** 2024-12-21

**Authors:** Alexander I. Balitskii, Andriy M. Syrotyuk, Valerii O. Kolesnikov, Valentina O. Balitska, Ljubomyr M. Ivaskevych, Maria R. Havrilyuk

**Affiliations:** 1 Department of Strength of the Materials and Structures in Hydrogen-Containing Environments, Karpenko Physico-Mechanical Institute, National Academy of Sciences of Ukraine, 5 Naukova Str., 79601 Lviv, Ukraine; syrotyuk@ipm.lviv.ua (A.M.S.); kolesnikov@ipm.lviv.ua (V.O.K.); ivaskevich@ipm.lviv.ua (L.M.I.); gavriluk@ipm.lviv.ua (M.R.H.); 2 Department of Mechanical Engineering and Mechatronics, West Pomeranian University of Technology in Szczecin,19 Piastow Av., 70-310 Szczecin, Poland; 3 Department of Professional Education, Taras Shevchenko National University of Lugansk, Kovalya Str. 3, 36000 Poltava, Ukraine; 4 Department of Physics and Chemistry of Combustion, Lviv State University of Life Safety, 35 Kleparivska, 79000 Lviv, Ukraine; vbalitska@yahoo.com

**Keywords:** hydrogen charging, 38KhN3MFA steel, turbine generator rotor shaft, turning, chips, cutting products, oxide, computer simulation

## Abstract

Rotor shafts are the most heavily loaded and accident-prone parts of powerful turbine generators, which are cooled using hydrogen. To eliminate damage sustained during operations, repair work was carried out, including the removal of defective parts, surfacing, and turning. This study tested the machinability of the rotor shaft using prototypes made from 38KhN3MFA steel. A section of the shaft was degraded due to prolonged operation (250 thousand hours), and compared to the central part, a decrease in the average grain size from 21.57 μm to 12.72 μm and an increase in the amount of hydrogen absorbed during operation from 2.27 to 7.54 ppm were observed. With the frequency of dry turning increasing from 200 to 315 RPM, the chips changed their form from mostly rectangular with linear dimensions of 10 to 20 mm to large spiral rings with a diameter of 15 to 20 mm and a length of more than 50 mm. Cracks of 1 to 4 mm in length were found in most chip particles at both rotational speeds. Increasing the rotational speed from 200 to 315 and up to 500 RPM led to the formation of an oxide film on the surface of the specimens, as evidenced by the appearance of oxygen during local analyses of the elemental content on the chip surface. The saturation of specimens by hydrogen gas led to the formation of finer chips compared to the non-hydrated material, and the roughness of the machined surface increased at hydrogen contents of 6 and 8 ppm. In both dry and coolant cutting operations, surface roughness reflects the degradation of the rotor shaft or experimental prototypes due to hydrogenation, which can be used to diagnose the condition of the rotor after long-term operation.

## 1. Introduction

Turbogenerator (TG) and steam turbine rotor shafts are manufactured from CrMoNiV steels (25Kh1M1FA, 20Kh3MVF, 34KhN3MA, 38KhN3MFA, 34NiCrMoV14-5, and 35NiCrMoV12-5) [1,2,3,4]. Depending on their intended use, as a result of heat treatments, they have a microstructure consisting of metal matrices of sorbitol, troostite, or bainite together with complex carbides, intermetallics, MnS, etc. The presence of vanadium increases the fine-grained microstructure of the surface layer, while the presence of bainite in the central part of the rotor shaft provides a good combination of strength, ductility, and impact strength [1,2,3]. During the long-term operation of power equipment, under the complex influence of factors such as changes in speeds, loads, temperatures, and exposure to technological media [5,6], including hydrogen-containing materials [4,7,8,9,10,11], irreversible changes are made to the structural and phase composition. At the same time, the structure of the steel undergoes different changes in various parts of the rotor, which is reflected in the microhardness, which differs by 20% [2].

Surface defects that occur during machining range from nano- to macroscale, causing microstructural, mechanical, and chemical effects that shape the performance properties and influence the operating conditions of the rotor system [12] and its turning processes [7,13,14,15,16,17]. The evolution of the microstructure and chemical composition due to the machining of materials causes integrity defects to form in the near-surface regions, which can significantly change the resistance to various forms of corrosion and characteristics such as fatigue and wear [18,19,20,21]. The surface and subsurface layers in chromium–molybdenum–vanadium steel formed during machining have increased hardness and residual compressive stresses of up to −1800 MPa, and they affect operational properties [22].

A study that focused on the effect of cutting temperatures and surface cooling rates on the microstructure and properties of so-called induced white layers during the turning of martensitic- and bainitic-hardened steel AISI 52100 showed that white layers formed both above and well below the austenitic transformation temperature of the original austenite Ac1, which was about 1023 K [23]. The surface cooling rate during turning reached 10^4^–10^5^ °C/s for cutting speeds from 30 to 260 m/min, regardless of whether the microstructure under study was bainite or martensite. For the orthogonal cutting of AISI 52100 steel, it was found that the white layer formed as a result of the rapid transformation of austenite and the quenching process, and the dark layer formed as a result of the tempering process [24]. Plastic deformation promotes the austenite transformation of the white layer and the tempering process of the dark layer, and it plays a role in improving the grain of both the white and dark layers. The rapid heating process caused by the cutting process causes austenite transformation and increases the austenite nucleation rate. Plastic deformation provides the driving force for the phase transformation, which can accelerate the formation of the white layer. At the same time, defects, such as dislocations, formed as a result of plastic deformation, which crushed the sub-grains in the white layer. Heat transferred to various depths, and stresses caused by plastic deformation provide the driving force for dynamic reduction and dynamic recrystallization in the dark layer; sub-grains in the dark layer are segmented by the movement of dislocations. The feed rate and cutting-edge radius affect surface integrity and fatigue endurance in turning 34CrNiMo6 steel [25]. The results show that the effect of residual stresses on fatigue life is more pronounced than the effect of surface roughness.

The structural-phase state of alloys significantly affects their properties and machinability [26,27,28,29,30,31,32]. Three different microstructures were obtained via controlled thermomechanical treatment, namely ferrite–pearlite, hardened martensite, and the ferrite–bainite–martensite of microalloyed vanadium steel 38MnSiVS5 [29]. The ferrite–bainite–martensitic microstructure shows better machinability due to its good surface texture, and the ferrite–perlite microstructure with the lowest strength is characterized by lower cutting forces. The experiments involved three different steel grades: C45 carbon steel, 37MnSi5 microalloy steel, and 30CrV9 low-alloy steel, which were subjected to four different types of heat treatment: normalizing annealing, mild annealing, quenching, and tempering followed by tempering. These tests showed that a homogeneous microstructure is the most important factor when it comes to the cutting quality of steel; the greater the difference in the hardness of structural components with heterogeneous microstructures, the higher the value of roughness [30].

The important informative factors that allow us to assess the micromechanisms of alloy fractures during machining include the study of chip morphology [31,32,33,34,35,36,37,38]. Two types of 42CrMo4 steel bars were studied, and each bar was subjected to a different heat treatment process to avoid any possible influence that chemical composition may have on the turning process [39]. The steel modifications investigated exhibited substantial differences in ductility (about 28.5% for the bainitic grade and 55.9% for the martensitic grade). The experiments performed in the current study show that the microstructure had no influence on the chip length when steel ductility was similar. The chips produced during the turning of 42CrMo4 exhibit regularly spaced serrated teeth above certain cutting speeds, the value of which depended on the microstructure. These teeth are formed due to the adiabatic nature of the shear process. The frequency of these serrated teeth increased with cutting speed, and each time their values approached the value of the natural frequency of the system, chatter occurred, and high forces were registered [39]. During the machining of high-strength steel, a correlation was found between the evolution of the microstructure and the quality and performance of the treated surface layer [40]. The relationship between the microstructure and the performance of the machined surface was explored by establishing a cutting model. The results show that the treatment forms a gradient microstructure, which significantly improves the performance characteristics. According to the test results, treatment significantly increases the hardness of the top layer of the high-strength alloy steel while increasing its toughness, which is the result of the combined effect of fine-grained hardening and dislocation hardening caused by the above treatment process. Thus, the microstructure of machined surfaces is crucial to the overall performance and service life of mechanical structures [35,36,37,38,39,40,41].

Wear products can also act as identifiers of the processes of destruction and the wear of parts [11,12,42,43,44,45,46,47,48,49]. Both in machining conditions and during friction and wear, common tribological concepts are often used to explain the physical nature of fractures [48,49,50,51,52,53]. In order to reduce the number of experiments, computer modeling has become widespread in recent years in terms of material properties [54,55,56,57,58,59,60], machining [61,62,63,64,65,66,67], and the calculation and visualization of chip formation [68,69,70,71,72,73]. As a rule, in most works, fractography and chip formation conditions are first studied, and only then is computer modeling performed [74,75,76,77,78,79,80]. For example, Kouadri et al. [81] discuss the mechanisms of chip formation during cutting operations. Some experiments characterizing the morphology and microstructure of chips are presented, as well as a study of chips at high loads. The mechanisms of chip segmentation during cutting are analyzed. The influence of cutting conditions on cutting forces is considered. As a result, the phenomenon of chip segmentation correlates with cutting forces.

Modern turbine generator rotors are cooled using hydrogen. It is known that elements of metal structures undergo changes in physical and mechanical characteristics during operation in hydrogen-containing environments. Hydrogen, penetrating into the metal through its surface, affects the plasticity, yield strength, and strength of the metal, and it can also contribute to plastic deformation in the initial phase [4,9,10,82,83,84,85]. Therefore, it is necessary to monitor changes in the properties of rotor steel during operation, considering the embrittlement effect of hydrogen. One of the methods for diagnosing its condition is to observe the fractography of the chips obtained from long-operated hydrogenation during repair work operations and the emergency rotor shaft. Globally, such work was performed by us for the first.

The aim of this work is to study structural transformations on the rotor shaft surface as a result of long-term operations and to compare the morphology of chips formed during the turning of shafts and the hydrogenated and non-hydrogenated prototypes of 38KhN3MFA steel.

## 2. Materials and Methodology

**Figure 1:**
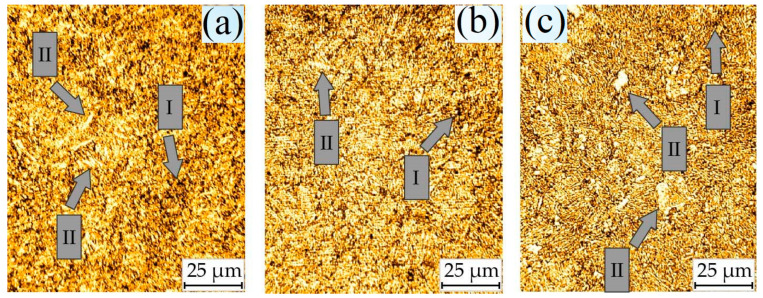
Microstructures of 38KhN3MFA steel specimens after tempering at 923 (**a**), 953 (**b**), and 1023 K: (**c**) I—sorbite colonies; II—carbides.

**Figure 2:**
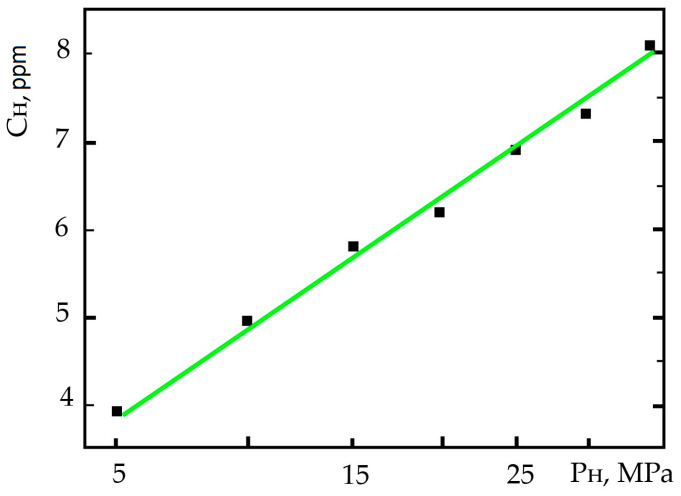
Dependences of the content of occluded hydrogen C_H_ on the hydrogenation pressure P at a temperature of 530 K for 10 h.

The chemical composition and properties of steel specimens are given in [7,9]. Specimens of 38KhN3MFA steel, which were hardened in oil from a temperature of 1123 K (holding time for 1 h) after tempering at 923, 953, and 1023 K for 2 h and cooling in air, were studied. Photographs of the microstructure of the specimens [9] are shown in Figure 1. The mechanical properties of five-fold cylindrical specimens with a working part diameter of 5 mm and a stretching speed of 1 mm/min are shown in Table 1; they were situated in a room-temperature environment. The specimens were saturated with hydrogen in a gaseous state using a special installation [9]. The exposure of specimens to hydrogen leads to hydrogen occlusion, and its content increases in proportion to the square root of the pressure (Figure 2), which is in good agreement with the known patterns of steel hydrogenation [86].

Sorbitol is a highly dispersed type of pearlite and comprises a eutectoid mixture of ferrite and cementite. The hardness, strength, and impact strength of sorbitol are higher than those of perlite. In terms of dispersion and hardness, it occupies an intermediate position between pearlite and troostite. The inter-plate distance in sorbite is 0.2 microns (in pearlite, 0.5–1.0 microns). Sorbitol is formed as a result of the decomposition of austenite at temperatures around 923 K during cooling (so-called quenching sorbite) and from martensite during tempering (tempering sorbite). With an increase in the tempering temperature, both the strength and ductility of steel decrease (Table 1), which is due to the enlargement of structural components, particularly an increase in the size of carbides (Figure 1c).

**Figure 3:**
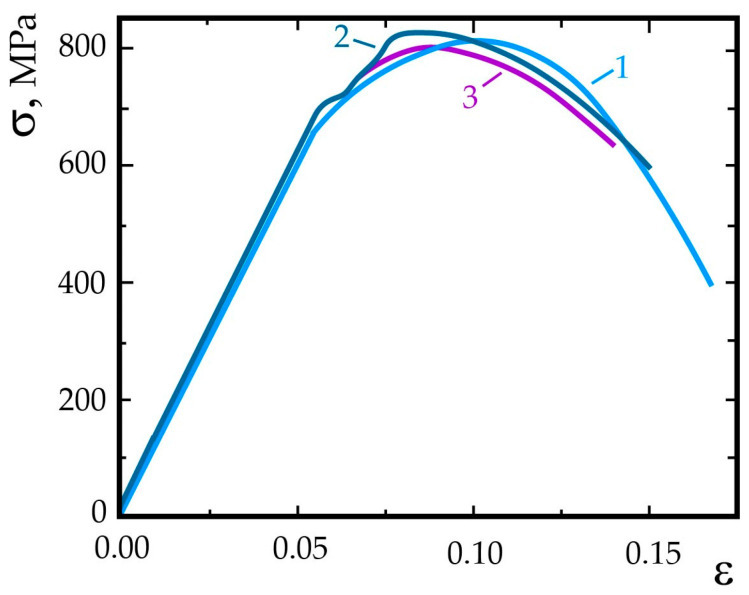
Stress–strain diagrams of rotor steel specimens after tempering at 1023 K in the air (1), in hydrogen at a pressure of 10 MPa (2), and in hydrogen at a pressure of 10 MPa after previous hydrogenation (C_H_ = 8.1 ppm) (3).

Hydrogen practically does not degrade the strength properties and significantly reduces plasticity characteristics (Table 2 and Figure 3). At a pre-absorbed hydrogen content of 8 ppm, its effect is manifested during tests in the air, and at a hydrogen environment pressure of 10 MPa, the properties of hydrogenated and non-hydrogenated samples are almost identical (Table 2), which is consistent with the patterns of the hydrogen embrittlement of steels with a volume-centered cubic lattice under the influence of external and internal hydrogen [82,86].

Full-scale studies were carried out in turning specimen conditions using 38KhN3MFA steel on a lathe-screw cutter (YANGTUO CK6140X1000 manufactured by Shandong Yangtuo CNC Machine Tool Co., Ltd. (located in Zaozhuang, China)). A VK-8 penetrating cutter was used; the machine speed was 200, 315, and 500 RPM; the loading rate was 0.1 mm.

Macrophotographs of the cutting products were taken with a Canon SX100 IS digital camera (Canon Company, Tokyo, Japan) equipped with a 10× optical zoom lens and a PowerShot SX100 IS image stabilizer. Microstructure photographs were taken using a microscope with a maximum magnification of 1000×. A microscope was used to take more detailed pictures of the cutting products: Zeiss Stemi 2000 (Carl Zeiss Company, Jena, Germany)—C Stereo Microscopes and a SIGETA digital camera (Industrial color digital camera UCMOS 1300, 1.3 MP and SIGETA International Color Digital Camera MCMOS 5100 5.1 MP.1) with a maximum resolution of 150 microns. The accuracy of the measurements was confirmed by the number of experiments, namely, from 3 to 5 experiments were performed at the same loads and speeds, which allowed us to obtain chips with comparable morphology and size. The chips were measured using a microscope with the appropriate software (Computer complex. ToupTek ToupView 3.7) using a scale setting that corresponded to 1 cm divisions. Local analysis for the determination of chemical elements was performed on an EVO-40XVP electron microscope (Carl Zeiss Company, Germany) with an INCA Energy 350 microanalysis system.

## 3. Results and Discussion

### 3.1. Analysis of Changes in Shaft Microstructure Parameters That Occurred During Operation

**Figure 4:**
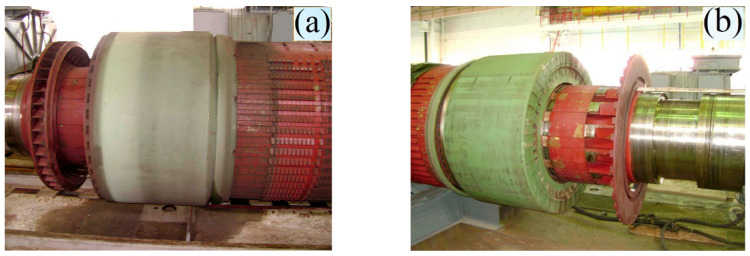
Damaged (**a**) and repaired (**b**) turbogenerator rotor shaft before transportation to the NPP turbine hall.

The rotor shaft consists of a middle active part (barrel) and two shanks. It is characterized by a single forging of steel from the high-strength category. To manufacture the shaft, an ingot about twice as large is used, which is the limit value for modern metallurgy. The large size of the shaft and the peculiarities of its operation lead to various operating situations that require repair work. In Figure 4, the appearance of the rotor shaft before and after repair work is shown.

**Figure 5:**
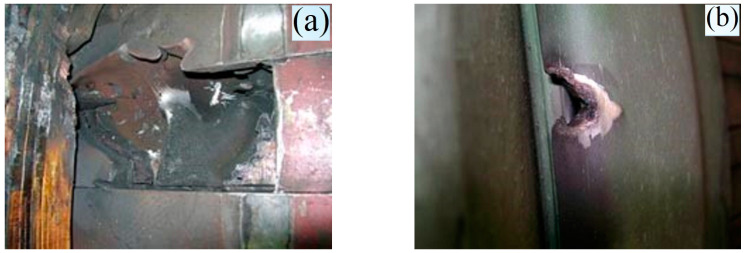
Damaged TGV-1000 shaft (after winding short circuit) in the hydrogen sealing zone. (**a**)—rotor body, (**b**)—retaining ring.

After 250,000 h of turbine unit operation, the hydrogen seals of the rotor shaft on the turbine side were destroyed. As a result of the friction of the fragments of the hydrogen seal against the tangential ridge, the shaft was partially destroyed (Figure 5). The boss bearings melted. The metallographic inspection showed that the rotor shaft metal in the hydrogen seal zone changed compared to the rotor shaft’s metal, which did not come into contact with the hydrogen seal.

The repair technology consisted of the fact that an insert made of the same material weighing up to 20 kg is mounted in place of the damaged material. “Sealing” is only part of the technological process of problem solving. Most attention is paid to the method of its installation on the rotor barrel and the proposed design of a special lock. The analysis of the durability of the structure under significant centrifugal forces arising during operation was taken into account.

After repairs, the “rotor-retaining ring” unit was connected to the grid on 28 August 2012, and up until the present time (2024), a reserve rotor has been used for safety (with controlled dimensions (Table 3)).

**Figure 6:**
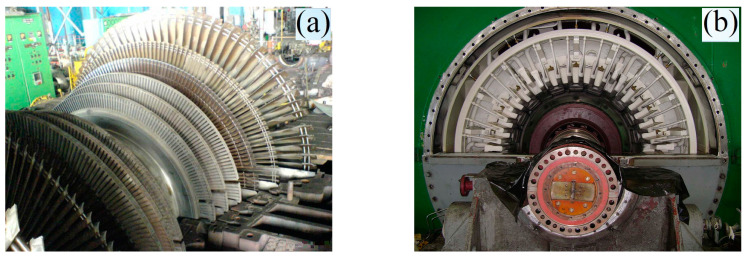
Rotor shaft of the turbine unit with damaged blades (**a**) and disassembled bearing (**b**).

Figure 6 shows the machine shop where the steam turbine rotor shaft is machined. In some cases, machining does not require dismantling the rotor shaft, but it takes place on-site in the machine shop.

**Table 3:** Rotor section dimensions (diameter designations are shown in Figure 7).

Section	D1	D2	D3	D4	D5	D6	D7	D8	D9	D10	D11	D12	D13
I–I (0°)	1100.12	884.80	649.61	599.19	677.16	578.72	677.30	677.14	578.74	677.05	579.07	677.18	679.51
II–II (120°)	1100.16	884.90	649.69	599.19	677.18	578.70	677.34	677.15	578.72	677.08	579.10	677.19	679.52
III–III (240°)	1100.20	884.85	649.63	599.16	677.20	578.71	677.32	677.14	578.73	677.12	579.08	677.18	679.51
Average	1100.16	884.85	649.63	599.18	677.18	578.71	677.32	677.14	578.73	677.08	579.08	677.18	679.51

**Figure 7:**
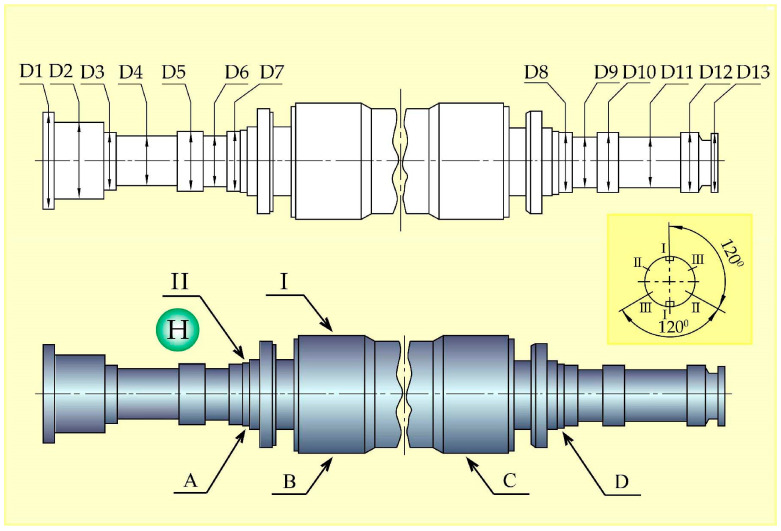
Schematic of the rotor shaft. Test area I—central part, which corresponds to the initial condition; Test area II—hydrogen seal. H—hydrogen, A, B, C, D—microhardness measurement locations (Table 4), D1–D13—rotor section diameters (Table 3).

**Figure 8:**
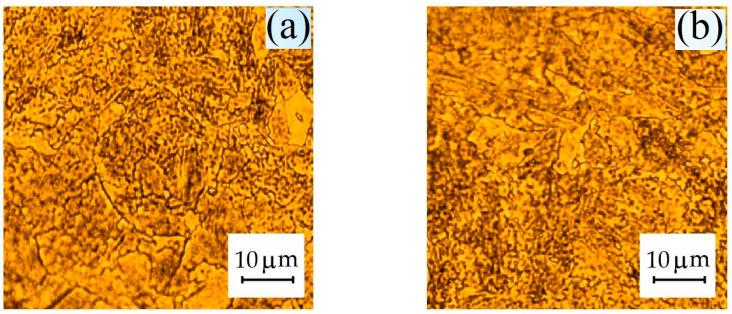
Microstructure on the rotor shaft surface (**a**,**b**). The initial conditional state from the rotor shaft surface (I—(**a**)). The state in the hydrogen seal zone (II—(**b**)).

The dimensions of the individual sections of the rotor shaft are shown in Table 3, and the conceptual diagram of the TG shaft is shown in Figure 7. Arrow I shows the area in which significant degradation processes are not recorded, and the microstructure of this zone corresponds to the initial conditional state (Figure 7 and Figure 8a). Arrow II schematically shows the place of destruction of the hydrogen seal, where microstructural changes were recorded (Figure 7 and Figure 8b). Here, in the zone of maximum hydrogen exposure, an increase in the microhardness of the shaft was found (Table 4).

**Figure 9:**
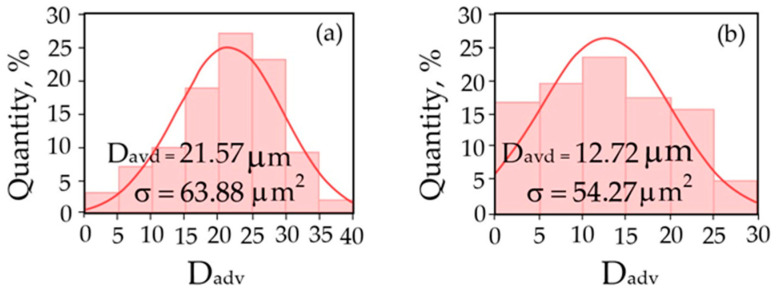
Histograms with the distribution of inclusion sizes (**a**,**b**). Initial condition of the rotor shaft surface (I—(**a**)); state in the hydrogen seal zone (II—(**b**)).

**Figure 10:**
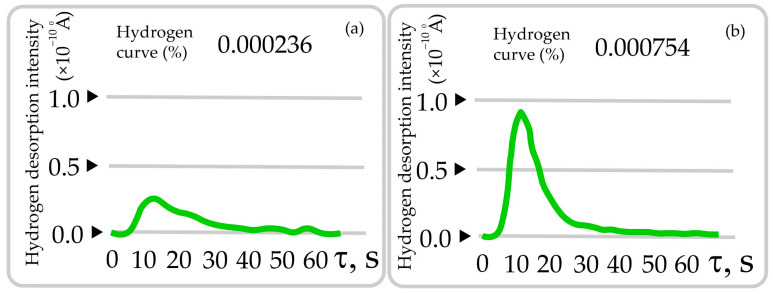
Amount of hydrogen in the chips (**a**,**b**). Initial conditional state of the rotor shaft surface (I—(**a**)); state in the hydrogen seal zone (II—(**b**)).

For site I, the average grain size was D = 21.57 μm, and the variance was σ = 63.88 μm^2^; for site II, the average grain size was D = 12.72 μm, and the variance was σ = 54.27 μm^2^. The largest average values of the data shown in the histograms (the central columns through which the maximum (extremum) of the normal distribution 2D RVE passes) correspond to 6, 7, and 8 (Figure 9a) and 9, 10, and 11 points of grain size (Figure 9b) according to DSTU 8972:2019 [87]. After machining and obtaining chips from areas I and II, the amount of hydrogen was determined to be 2.27 and 4.72 ppm (Figure 10a,b).

**Figure 11:**
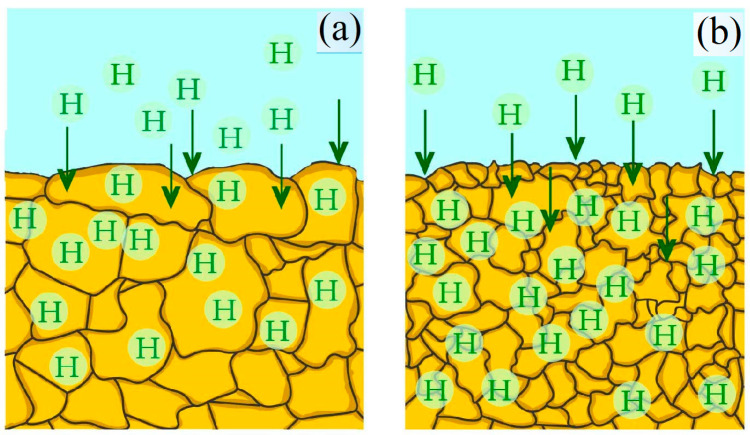
Scheme of microstructure: the initial conditional state of the rotor shaft surface (I—(**a**)); the state in the hydrogen seal zone (II—(**b**)).

Figure 11a,b show a schematic diagram that considers a scenario where a larger amount of hydrogen is fixed in the finer microstructure of the rotor shaft. During long-term operation, the rotor shaft comes into contact with a hydrogen-containing medium that cools the rotor shaft, and due to newly formed defects and damage due to the presence or formation of a thinner microstructure, the hydrogen saturation of the surface and near-surface layers occurs. 

Thus, during operation, under the influence of loads and the hydrogen environment, the rotor shaft undergoes structural changes that must be considered during its further exploitation. Since surface changes affect chip formation during turning [8,11,25,28], this paper considers the possibility of assessing the condition of the shaft’s surface by analyzing the generated chips and developing recommendations for shaft repair work based on the comparative studies results of the shaft and prototypes made from 38KhN3MFA steel.

### 3.2. Classification and Analysis of Chips of the Rotor Shaft and Experimental Prototypes

**Figure 12:**
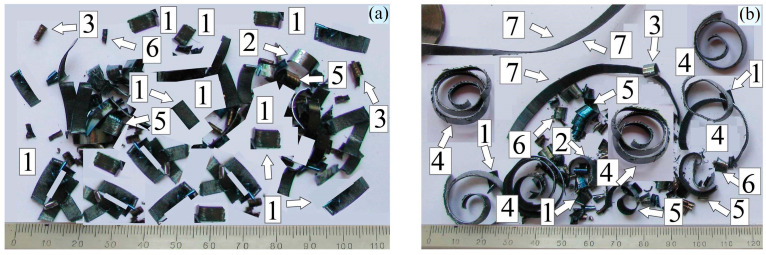
Appearance of turning products obtained under dry cutting conditions: at 200 RPM (**a**), 315 RPM (**b**). Numbers 1–7 indicate different types of chips, the appearance and size of which are indicated in the caption under Figure 13.

**Figure 13:**
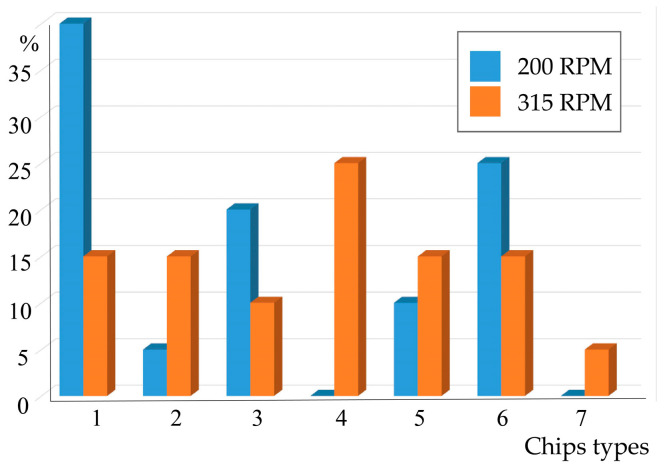
Distribution of chips in percentages according to the developed classification at speeds of 200 RPM and 315 RPM: 1—rectangular and large (predominantly 10–15 mm in length); 2—rolled into a half-ring with a radius of 5–7 mm; 3—completely rolled (compact) (radius up to 5; predominantly 1–3 mm); 4—rolled into rings (from 7 mm—mainly 15–20 mm); 5—semi-rolled with linear dimensions of 10–15 mm; 6—small broken chips of less than 2.0 mm; 7—large chips over 50 mm in length.

The appearance of 38KhN3MFA steel cutting products after machining is shown in Figure 12, and the distribution on the diagram according to the developed classification is shown in Figure 13. The following types of chips were identified: 1—rectangular with linear dimensions within 10–15 mm. Their number is more than 35% for cutting conditions of 200 RPM. At 315 RPM, their number decreases, but chips of type 4 and 7 appear, the former having the form of large coiled spiral rings with a diameter of 15–20 mm, and the latter having a large ribbon shape with a chip length of more than 50 mm. There are also several types of small chips, and their description is available in publication [11].

**Figure 14:**
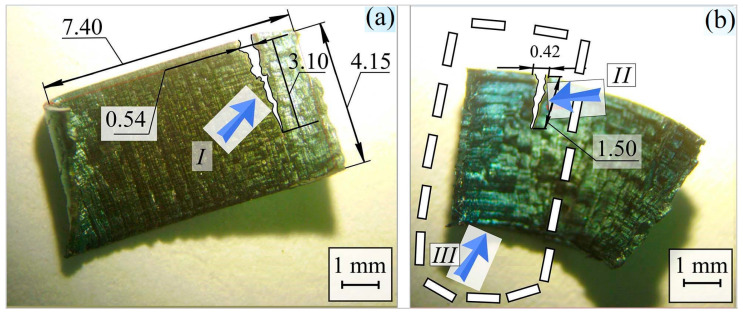
Appearance of chips obtained under dry cutting conditions: 200 RPM (**a**) and 315 RPM (**b**). A rectangular particle (4.15 by 7.40 mm) with a crack indicated by arrow I. dotted frame—colors of the variability.

Figure 14 shows the appearance of the cut products: a rectangular particle (4.15 by 7.40 mm) with a crack indicated by arrow I (Figure 14a). The length of the crack is 3.10, and the width of the crack at the beginning is 0.54 mm. On the surface of the particle, the colors of variability are almost invisible. Figure 14b shows an image of a particle formed under cutting conditions at 315 RPM. Arrow II indicates a crack with a width of 0.42 mm and a length of 1.5 mm; III indicates the left side of the chip, which shows the colors of the variability. The width of the cloves in this area is 0.37 mm, and for the rest of the chip, it is 0.32 mm.

**Figure 15:**
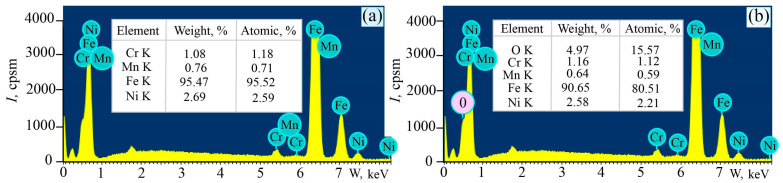
Results of local analysis of the element content on the chip surface after turning at 200 (**a**) and 315 RPM (**b**).

The brightness of the color variation depends on the thickness of the oxide film formed and the wavelength of light that hits the surface of the material. It is important to note that the metal’s melting point differs for each individual alloy and metal type. Therefore, there are a large number of tables and lists of color and temperature correlations. The part that has irregularities also has a dense film. The colors of variability on the surface of the particle are blue and dark green. For carbon steel, this corresponds to a surface temperature of 583–603 K [88,89]. Increasing the rotational speed from 200 to 315 RPM results in the formation of an oxide film on the surface of the specimens, as evidenced by the appearance of oxygen during local analysis of the elemental content on the chip surface (Figure 15). An even more significant increase in temperature and, consequently, surface oxidation occurs at rotational speeds up to 500 RPM, so this turning mode is not recommended. The computer calculations below confirm the increase and change in the turning temperature of the workpiece.

**Figure 16:**
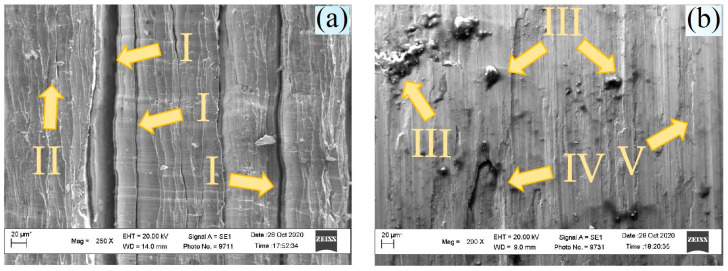
Internal surface of chips (photo of chips from Figure 14a) at 200 RPM (**a**) and 315 RPM (photo of chips from Figure 14b) (**b**).

Figure 16 shows photos of the inner surface of the chip (corresponding to the chips shown in Figure 14). At 200 RPM, chips with stripes and vertical cracks were formed: position I (Figure 14a) had areas with small welded particles; position II had chips with welded particles of the material at 315 RPM (position III). The welded particles are larger in size and have a larger area compared to those shown in Figure 16a. Material delamination (position IV) and areas with microrelief (position V) were also observed, i.e., two different competing micromechanisms of fracture during chip formation were recorded. Thus, when the temperature rises, conditions are created for welding the material, which is accompanied by an increase in roughness.

**Figure 17:**
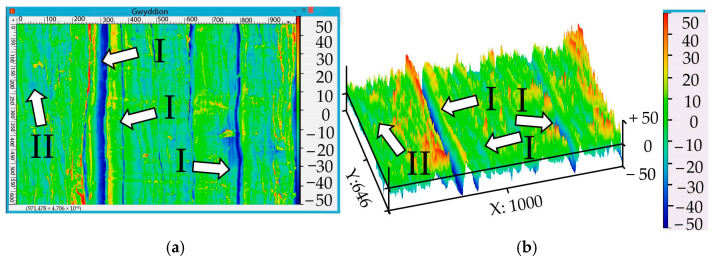
Two-dimensional (**a**) and three-dimensional (**b**) reconstruction of the chip area presented in Figure 17a.

**Figure 18:**
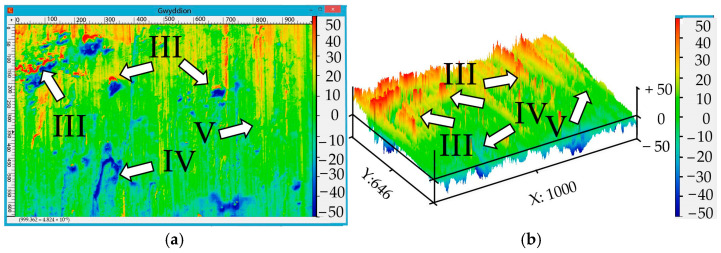
Two-dimensional (**a**) and three-dimensional (**b**) reconstruction of the chip area presented in Figure 9b.

Images of 2D and 3D computer reconstructions of the chip’s surface are shown in Figure 17 and Figure 18. In order to summarize the results of the roughness scale, the scale’s size ranges from 0 to 100 units. As shown in Figure 14, in the locations of vertical cracks (position I), a maximum depth from 0 to −50 units can be observed. On the left, a section along the entire crack with a height of up to 50 units was recorded. On the right part of the image, near a narrower crack, there is no such section. However, on the right, in the most extreme part, peaks with a height of up to 45 units were recorded. Area (II) has a gentle microrelief with elevations from −15 to 15 units.

In Figure 18, position III indicates areas with heights ranging from 20 to 50 units that were most likely formed during the welding of the material. The elongated depression (IV) has a depth fluctuation from −15 to −35 units. The area with a smooth microrelief (V) is characterized by heights from −5 to +15 units.

**Figure 19:**
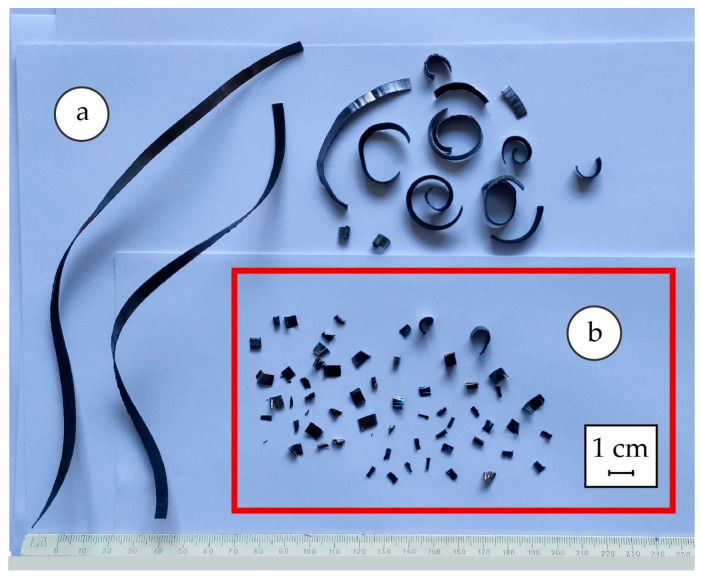
Chips formed during dry cutting at 315 RPM: before hydrogenating (**a**); after hydrogenating (**b**).

The presence of absorbed hydrogen in steel at amounts of 6–8 ppm contributes to the formation of fine chips regardless of the turning frequency (Figure 19). The macroimages of hydrogenated chips are shown in [7]. They are characterized by a significant number of cracks compared to the chips that were separated from non-hydrogenated specimens. The use of computer vision technology allows us to determine the presence of such damage and draw a conclusion about the condition of the material.

The formation of a larger number of cracks in the hydrogeneted material can be attributed to the influence of hydrogen penetrating into the surface and subsurface layers. The temperature increase, intensification of plastic deformation, and presence of pores in the material affect the state and distribution of hydrogen. Molecular hydrogen accumulates in the pores and creates high pressures that contribute to steel embrittlement. The presence of hydride-forming elements can contribute to the retention of atomic hydrogen in steel [90]. Thus, a thorough diagnosis and fractographic analysis of the chips allow us to draw conclusions about the formation of chips under the complex influence of factors.

**Figure 20:**
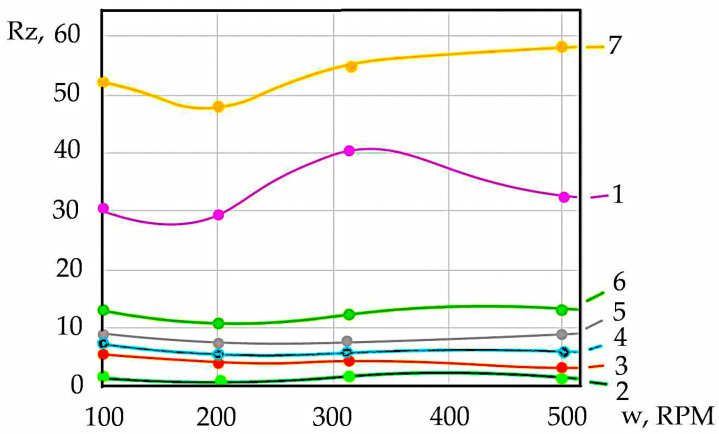
Graph of surface roughness: 1 and 2—conditionally initial condition; 3, 4, and 5—experimental prototypes after gas hydrogenation: hydrogen concentration 2, 4, and 5 ppm, respectively; 6 and 7—specimens from the degraded part of the rotor shaft (6 and 7.54 ppm, respectively). Cutting conditions: 1 and 7—dry cutting; 2–6—cutting with coolant.

The surface roughness after turning specimens from 38KhN3MFA steel depends on the rotational speed (Figure 20). It is recorded that at 100 RPM, the roughness is greater than at 200 RPM, which is explained by the fact that under such conditions, an outgrowth formed between the cutter and the workpiece, which interfered with finishing. An increase in the temperature in the cutting zone at 315 RPM contributed to an increase in roughness, but further increases in speed led to a more uniform removal of workpiece material and a decrease in roughness (curves 1, 2, 3, 4, 6, and 7). This pattern was particularly clear under dry cutting conditions for the specimen with ferrite–pearlite microstructure (curve 1). The highest roughness was observed for the specimen cut from the degraded part of the shaft (curves 5 and 6). This can be explained by the fact that during the interaction of the surface and subsurface layers of the rotor shaft during operation, complex physical and chemical processes are reflected as a result of long-term processes that lead to structural changes. For example, for turbine unit shafts, it was recorded that during long-term operation (up to 250 thousand hours), the surface hardness of the rotor shaft decreases from 290 HB to 250 HB. It was recorded that in the microstructure of the shaft, the amount of cementite decreased from 87% to 62%, and the proportion of free ferrite increased from 5% to 20% over 250 thousand hours of operation. The average microhardness of ferrite decreased from 1.9 GPa to 1.5 GPa. An increase in the content of alloying elements in carbides was recorded: Cr and V—by 1.15–1.6 times; Mo—by 2.2–2.8 times [7,91,92]. The hydrogen saturation of the specimen with a sorbitan microstructure at a hydrogen concentration of 2 ppm reduced the surface roughness compared to the non-saturated specimen. At such lower hydrogen concentrations, the “plasticising effect” of hydrogen in the steel matrix was observed [85,93,94]. For specimens with a hydrogen concentration of 6 and 8 ppm, the microhardness (Table 4) and roughness (Figure 20; curves 4 and 5) were higher than for the unhydrogenated sample, indicating the brittle nature of the hydrogen effect. Also, the embrittling effect of hydrogen [7,9,82,85,86] is confirmed by a decrease in the values of δ and ψ after the saturation of the specimens with hydrogen (Table 1 and Table 2).

Thus, during machining, the processes of forming micro- and macro-geometric parameters and the stress–strain state of the surface and subsurface layers of the part continue. Hydrogen localizes and intensifies the processes of plastic deformation and facilitates fracture by penetrating into the formed microcracks. Active radicals interact with the juvenile surface of the workpiece and the tool through chemisorption, reducing energy consumption during turning, and “nanofluids” and lubricants are used to improve machining and to cool the tool during technological operations [11,43,44,45,46,47,48,49]. In both dry and coolant cutting, the surface roughness reflects the degradation of the rotor shaft or experimental prototypes due to hydrogenation, which can be used to diagnose the condition of the rotor after long-term operation. Thus, taking into account the embrittlement effect of hydrogen on the properties and formation of chips allows for a more reliable assessment of the change in the operational characteristics of the rotor shaft.

## 4. Conclusions

Comparative studies of the turning products of a long-term operated rotor shaft and experimental samples of rotor steel 38KhN3MFA were carried out at turning speeds from 100 to 500 rpm. The effect of absorbed hydrogen on the surface roughness and the appearance of the formed chip particles was evaluated. The parameters of the microstructure corresponding to area I—the central part of the rotor shaft (initial conditional state)—and area II—the location where the hydrogen seal and bearing were destroyed—were analyzed. In the area degraded due to long-term operation (250,000 h), a decrease in the average grain size from 21.57 μm (area I) to 12.72 μm (area II) and an increase in the amount of hydrogen absorbed during operation from 2.27 (area I) to 7.54 ppm (area II) were observed.

Dry turning at 200 RPM mainly produces rectangular chips with linear dimensions of 10 to 15 mm. With an increase in frequency to 315 RPM, the number of large spiral rings with a diameter from 15 to 20 mm increases with a chip length of more than 50 mm. Cracks of 1–4 mm in length were found in most chip particles for both turning frequencies.

Increasing the rotational speed from 200 to 315 RPM leads to the formation of an oxide film on the surface of the specimens, as evidenced by the appearance of oxygen during local analysis of the elemental content on the chip surface. As the temperature rises, conditions are created for welding the material, which is accompanied by an increase in roughness. An even more significant increase in temperature and, consequently, surface oxidation occurs at rotational speeds up to 500 RPM, so this turning mode is not recommended.

The saturation of specimens with gaseous hydrogen led to the formation of finer chips compared to the non-hydrated material. At a hydrogen content value of 2 ppm, the roughness of the machined surface was less than that at 6 ppm and 8 ppm. The maximum roughness values were Rz = 48–58 for the material cut from the degraded part of the rotor. In both dry and coolant cutting, the surface roughness reflects the degradation of the rotor shaft or experimental prototypes due to hydrogenation, which can be used to diagnose the condition of the rotor after long-term operation.

## Figures and Tables

**Table 1:** Mechanical properties of 38KhN3MFA steel specimens under different heat treatment regimens [9].

Tempering Temperature, K	σ_u_ MPa	σ_y_ MPa	δ %	ψ %
923	940	790	21	67
953	890	750	18	48
1023	810	690	17	44

**Table 2:** Mechanical properties of 38KhN3MFA steel at room temperature in the air and under various hydrogenation conditions [7,9].

Temperature		The Conditions of the Tests	σ_u_ MPa	σ_y_ MPa	δ %	ψ %
Hardening, K	Tempering, K					
1133	913	air	950	800	17	51
1123	923	air	940	790	21	67
		air, C_H_ = 8 ppm	960	790	16	45
		hydrogen, 0.8 MPa	930	810	18	55
		hydrogen, 10 MPa	880	780	12	36
		hydrogen, 10 MΠa, C_H_ = 8 ppm	900	790	12	35
	953	air	890	750	18	48
		air, C_H_ = 8 ppm	900	740	14	31
		hydrogen, 0.8 MPa	890	740	14	36
		hydrogen, 10 MPa	880	760	11	28
		hydrogen, 10 MPa, C_H_ = 8 ppm	900	750	12	29
	1023	air	810	690	17	44
		air, C_H_ = 8 ppm	830	720	15	32
		hydrogen, 0.8 MPa	790	700	13	36
		hydrogen, 10 MPa	780	680	14	29
		hydrogen, 10 MPa, C_H_ = 8 ppm	800	700	14	28

**Table 4:** Hardness of the rotor sections (designations are shown in Figure 8).

Measurement Location	Hardness HB
A	285/291
B	230/243
C	238/251
D	257/270

## Data Availability

The original contributions presented in this study are included in the article. Further inquiries can be directed to the corresponding author.

## Nomenclature and Abbreviations

σ_u_	ultimate tensile strength (UTS)
σ_y_	yield strength (YS)
δ	elongation
ψ	reduction in area
C_H_	hydrogen concentration in specimens
P	hydrogen pressure
D	average grain size
RPM	revolutions per minute
TGV	turbogenerator with hydrogen cooling
HB	Brinell hardness
HELP	hydrogen-enhanced localized plasticity
HEDE	hydrogen-enhanced decohesion effect
